# Molecular Characterization of *Diaporthe* Species Associated With Hazelnut Defects

**DOI:** 10.3389/fpls.2020.611655

**Published:** 2020-12-11

**Authors:** Roberta Arciuolo, Carla Santos, Célia Soares, Giuseppe Castello, Nicola Spigolon, Giorgio Chiusa, Nelson Lima, Paola Battilani

**Affiliations:** ^1^Department of Sustainable Crop Production, Università Cattolica del Sacro Cuore, Piacenza, Italy; ^2^CEB – Centre of Biological Engineering, Micoteca da Universidade do Minho, University of Minho, Braga, Portugal; ^3^Soremartec Italia S.r.l., Alba, Italy

**Keywords:** fungi, *Diaporthe*, *Phomopsis*, hazelnut, rotten, molecular phylogeny, multi-locus sequence analysis, Turkey

## Abstract

Fungi of the genus *Diaporthe* have been reported as the main causative agent of hazelnut defects in the Caucasus area. This study aimed to define which fungal species are present in defective hazelnuts grown in Turkey and confirm the role of *Diaporthe* spp. Seven hazelnut orchards were selected, with each one located in a different Turkish Province (Düzce, Giresun, Ordu, Samsun, Sakarya, Trabzon, and Zonguldak), and hazelnuts were collected at early and full ripening. Fungal isolation and identification were performed at the genus level based on morphological characteristics. Several genera were isolated, with *Diaporthe* spp. being among the prevalent. This was the only genus with increasing incidence from early to full ripening, and incidence at full ripening was positively correlated both with internal (ρ = 0.86) and visible defects (ρ = 0.81), which confirmed its role as the key causative agent of hazelnut defects. The correlation of defect occurrence with rainfall, reported in previous study, was not confirmed, possibly due to the low defect incidence. A total of 86 *Diaporthe* monosporic strains isolated from Turkish hazelnut samples, together with 33 strains collected in the Caucasus region and 6 from Italy, were analyzed with a multi-locus phylogeny based on three genomic loci (ITS, *EF1*-α, and *tub*). The results showed that *Diaporthe* strains can be grouped into 7 distinct clades, with a majority of Turkish strains (95%) being placed into a single clade related with *D. eres*. These samples were organized into several sub-clades, which indicates the existence of genetically diverse sub-populations.

## Introduction

Hazelnuts (*Corylus avellana* L.) are cultivated worldwide in areas of mild climate and high humidity. When considering the worldwide production of tree nuts, hazelnuts are the fifth most highly produced nut,^1^ at 528.07 thousand metric tons that are directly consumed or processed. Turkey is the main grower, producing approximately 72.9% of the total world supply^2^. In Turkey, hazelnuts are grown in different provinces, mainly in the Black Sea area (Islam, 2018).

Several defects have been reported in hazelnuts, such as the presence of blemishes, areas of discoloration, or stains in marked contrast with the rest of the kernel (Teviotdale et al., 2002). The resulting defective kernels are not compliant with the quality standards required by the market^3^. The incidence of defects varies between 1 and 15%, depending on the year, weather conditions, and growing area^4^. Thus, hazelnut defects, defined as “rotten hazelnuts” by commercial evaluation, negatively impact kernel availability on the market as well as economics. The identification of the causal agents is critical in order to define and apply preventive actions, improve hazelnut yield and quality, and thus increase the market value.

*Diaporthe* spp. fungi appears as necrotic spots on kernel surfaces and causes internal browning that is visible after cutting the nut in half (half-cut). In a previous study in the Caucasus region, *Diaporthe* spp. was identified as the crucial genus involved in causing hazelnut defects. Only three strains were identified at the species level in the study, but this suggested that *D. eres* was responsible for the visible brown spots on the kernel surface and the internal discoloration observable after the nut was cut in half (Battilani et al., 2018). *D. eres* was also recently reported by other authors as associated with hazelnut trunk cankers in Oregon (Wiman et al., 2019), while *D. foeniculina* was mentioned by Guerrero et al. (2019) as causing black tip and necrotic spots on hazelnut kernels in Chile and *D. rudis* was detected in hazelnut kernels with visible mold in Oregon (Pscheidt et al., 2019).

*Diaporthe* species have been frequently reported as an important group of plant pathogenic fungi, non-pathogenic endophytes, or saprobes, and are related to diseases that occur in a wide range of economically important plants (Farr et al., 2002a,b; Crous, 2005; Udayanga et al., 2011; Gomes et al., 2013; Huang et al., 2015; Chepkirui and Stadler, 2017; Guarnaccia et al., 2018).

Since their discovery, *Diaporthe* spp. and their asexual stage *Phomopsis* spp. have been identified based on morphology and host association (Uecker, 1988; Ferreira et al., 2015). However, the association between host and species is not reliable within the *Diaporthe* genus as an identification criterion (Gomes et al., 2013; Udayanga et al., 2014a,b). It has been observed that the same *Diaporthe* spp. colonizes different hosts, and the co-occurrence of different species is commonly reported in the same host (Rehner and Uecker, 1994; Mostert et al., 2001; Guarnaccia et al., 2016; Guarnaccia and Crous, 2017). Additionally, many studies have recently claimed that morphology is generally not conclusive for identification at the species level due to the high complexity of the *Diaporthe* genus (Santos et al., 2010; Udayanga et al., 2011; Dissanayake et al., 2017a,b). Therefore, by using molecular approaches, substantial progress regarding the identification and characterization of emerging pathogens in the *Diaporthe* genus has been realized (Santos and Phillips, 2009; Diogo et al., 2010; Luongo et al., 2011; Udayanga et al., 2012a,b; Thomidis et al., 2013; Gao et al., 2017; Guarnaccia and Crous, 2017). Notably, among the various techniques, multi-gene phylogenetic species delineation is becoming the most effective instrument for taxonomic studies of fungi (Taylor et al., 2000; Dettman et al., 2003). Regarding *Diaporthe* spp., the taxonomy of the genus is especially based on ITS (internal transcribed spacer region of ribosomal DNA), *EF1-*α (translation elongation factor 1-alpha gene), *tub* (β-tubulin), and *cal* (calmodulin) loci sequences (Udayanga et al., 2012a; Gomes et al., 2013).

Based on this background, the objectives of this study were to (i) investigate the fungi associated with defective hazelnuts in Turkey, with a special focus on the role of *Diaporthe* spp.; (ii) characterize *Diaporthe* strains based on molecular techniques, using multi-locus phylogenetic species identification by means of ITS, *tub*, and *EF1-*α; and (iii) identify, at the sub-genus level, the *Diaporthe* strains isolated from hazelnut kernels.

## Materials and Methods

### Culture Media

Potato Dextrose Agar (PDA). Agar, 15 g; natural potato broth obtained from 200 g potato, dextrose, 10 g, double-distilled water, 1 L; streptomycin sulfate added during cooling, 0.15 g L^–1^. Water Agar (WA). Agar, 15 g; double-distilled water, 1 L. Streptomycin sulfate added during cooling, 0.15 g L^–1^. Malt Glucose Yeast Peptone (MGYP). Malt extract, 3 g; mycological peptone, 5 g; glucose, 10 g; yeast extract, 3 g; double-distilled water, 1 L.

### Molecular Biology Buffers and Solutions

Hexadecyl trimethyl-ammonium bromide 2% buffer (CTAB). Cetyl trimethylammonium bromide (CTAB), 2.0 g; 2 M Tris-HCl pH 8.4, 5.0 ml; 0.2 M ethylenediaminetetraacetic acid (EDTA), di-sodium salt, pH 8.0, 12.48 ml; NaCl, 8.18 g. The volume was adjusted to 100 ml with distilled water. 2 M Tris-HCl pH 8.0. Tris base, 12.114 g; distilled water, 50 ml. The pH was adjusted to 8.0 by adding 2 M NaOH. 0.2 M EDTA pH 8.0. Ethylenediaminetetraacetic acid, di-sodium salt, 3.7224 g; distilled water, 50 ml. The pH was adjusted to 8.4 by adding HCl. The solution was cooled to room temperature before making the final adjustments to the pH. 1% Agarose gel. Agarose, 1 g; Tris-acetate EDTA (TAE) 0.5× buffer, 100 ml; GreenSafe Premium (NZYtech, Lisbon, Portugal), 5.3 μL. 50× TAE buffer. Tris base, 242 g; acetic acid, glacial, 57.1 mL; 0.5 M EDTA pH 8.0, 100 mL; distilled water to 1 L. To make a 0.5× working solution, the concentrated stock solution was diluted at 1:100 dilution concentrated stock.

### Hazelnut Orchards and Meteorological Data Collection

**FIGURE 1:**
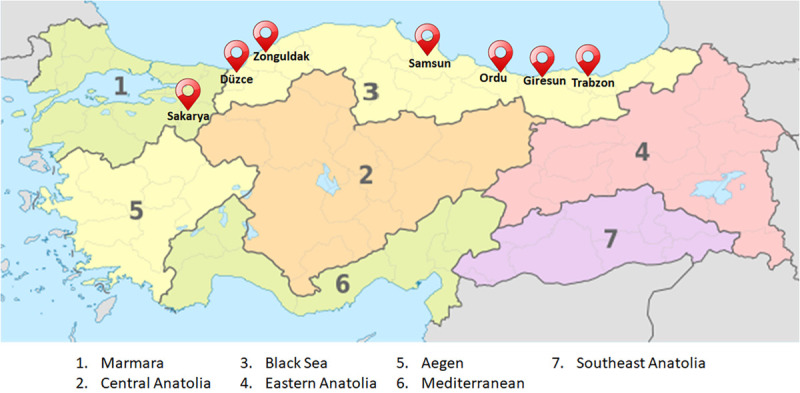
Distribution of the seven sampled hazelnut orchards located in different Turkish provinces (https://upload.wikimedia.org/wikipedia/commons/f/f9/Turkey_ %28regions%29%2C_administrative_divisions_-_Nmbrs_-_colored.svg adapted).

In 2017, 7 hazelnut-growing provinces in different Turkish locations were selected for this study (Figure 1). One orchard was considered in each province, for a total of 7 sampling points. Three orchards were located in hazelnut growing provinces in West Turkey (Düzce, Sakarya, Zonguldak), and the other orchards were located in 4 growing provinces in East Turkey (Giresun, Ordu, Samsun, and Trabzon). A mix of different hazelnut varieties were grown in the orchards sampled; Kara findik, Mincane, Çakildak and Foşa were reported in Sakarya, Düzce and Zonguldak, while Tombul, Palaz, Mincane, Çakildak, Foşa and Sivri in Samsun, Ordu, Giresun and Trabzon. Orchards were managed according to common hazelnut agricultural practices used in Turkey. At ripening, hazelnuts were hand harvested from the plant and dried on the ground for approximately one week; then, husks were mechanically removed and in shell drying continued on the ground until a final kernel humidity ≤6%. Wireless weather stations (Vantage Pro2^TM^ Plus^®^, Davis Instruments) were placed close to the orchards (approximately 500 m distance; the GPS coordinates are shown in Table 1), and hourly data consisting of air temperature (T, °C), air relative humidity (%RH), and rainfall (R, mm) were recorded from January 1st to August 30th in each orchard.

**TABLE 1:** Wireless weather station GPS coordinates of Turkish provinces selected for the study.

**Province**	**Latitude**	**Longitude**	**Location**
Düzce	40.887375	30.852033	West
Giresun	40.838950	38.635900	East
Ordu	40.842222	37.784444	East
Sakarya	40.986924	30.746225	West
Samsun	40.983461	36.669167	East
Trabzon	40.950185	39.991494	East
Zonguldak	41.103993	31.414249	West

### Hazelnut Sampling

Hazelnuts were collected approximately 45 days after setting, at early ripening (BBCH 81) and at full ripening (BBCH 89), according to Battilani et al. (2018). Each orchard was approximately 1,000–2,000 m^2^. In each orchard, 100 trees were selected along 2 diagonals of the orchard, and 30 hazelnuts per tree were randomly collected in order to obtain a total of 3,000 hazelnuts per sampling point and time. The nuts were shelled and observed for defects, both superficial and internal, the latter after the kernel was cut in half (Teserba GmbH, Rüti, Switzerland).

The hazelnuts were stored at 5°C under vacuum until they were delivered to the laboratory. Two hundred and seventy defective kernels were randomly selected from each of the 7 orchards (90 half-cut nuts, analyzed in triplicate), with some exception of smaller samples in BBCH81.

### Fungi Isolation and Identification

Hazelnuts collected at early and full ripening were processed using the same protocol. Half-cut kernels were washed with running tap water for 1 min, disinfected with 1% sodium hypochlorite solution for 1 min, rinsed 3 times in sterile double-distilled water, and then dried on sterile paper under a sterile hood. Half-cut kernels were plated in 90-mm diameter Petri dishes containing WA and incubated at 25°C, with a natural photoperiod, up to 21-day. The plates were viewed twice a week for fungal growth, and visible colonies were transferred to PDA dishes and incubated at 25°C, with a natural photoperiod, until the development of reproductive structures, for a maximum of 30-day. Morphological characterization was determined with the support of a stereomicroscope (Motic) with 40× magnification, and an optical microscope (Leitz labor lux D) at 500× magnification, following taxonomic keys to identify the isolates at the genus level (Raper and Fennell, 1965; Ellis, 1971, 1976; Pitt, 1979; Sutton, 1980; Rotem, 1994; Krol, 2005; Leslie and Summerell, 2007; Udayanga et al., 2011; Gomes et al., 2013; Visagie et al., 2013; Maharachchikumbura et al., 2014; Samson et al., 2014). When available, two to three well-separated colonies of *Diaporthe* spp. were selected from each replicate for further studies. They were subject to conditions that resulted in obtaining monosporic cultures according to Battilani et al. (2018). Colonies grown on PDA were transferred to tube vials containing WA and stored at 4°C until use.

Of the 125 *Diaporthe* spp. strains used in this study for molecular characterization, 86 *Diaporthe* spp. strains were collected in Turkey (this study), 33 in the Caucasus region (Battilani et al., 2018), and 6 were from Italy. The nuts were gathered using the same protocol as that used to collect hazelnuts in the Caucasus region. All 125 *Diaporthe* spp. strains were deposited in Micoteca da Universidade do Minho (MUM) culture collection, Braga, Portugal.

### *Diaporthe* spp. DNA Extraction

Following a previously described protocol (Rodrigues et al., 2009) with some modifications, all 125 monosporic *Diaporthe* spp. strains previously mentioned were inoculated into 5-mL tubes containing 3 mL of MGYP and incubated at 25°C for 14-day with rotation at 150 rev min^–1^. The mycelia were collected by filtration and stored at −20°C. Genomic DNA was extracted using the following protocol: 200 mg of filtrate was placed in a 2-mL tube containing 0.67 g glass beads (Sigma, 710-1, 180 μm) and 1 mL of CTAB buffer. FastPrep-24^TM^ 5G (MP Biomedicals) was used at speed 6 for 30 s to break down the cellular membrane. After centrifugation at 14,000 × *g* for 8 min, 650 μL of supernatant was transferred in a new 2-mL tube, 750 μL of sodium acetate (3 M, pH 5.5) was then added, and the liquid in the tube was gently mixed by inversion and subsequently incubated at −20°C for 10 min. Then, samples were centrifuged at 14,000 × *g* for 10 min, and 750 μL of supernatant was transferred to a new 2-mL tube containing 750 μL of 2-propanol, gently mixed, and incubated at room temperature for 1 h. Samples were centrifuged at 14,000 × *g* for 10 min, and the supernatant was decanted without disturbing the pellet, which was then washed with 750 μL ice-cold 70% ethanol. The ethanol was decanted, and the last step was repeated. Residual ethanol was removed by drying in a SpeedVac concentrator (SPDIIIV, Thermo Scientific).

DNA was dissolved in 50 μL of ultrapure water, and samples were placed in a water bath at 56°C for 2 h. Next, 1 μL of RNAse (10 mg/mL) was added, and the samples were placed again in a water bath at 36°C for 1 h. The DNA was subjected to quality assessment by electrophoresis (120 V/cm for 7 min plus 80 V/cm for 30 min) on 1% (w/v) agarose in 0.5× TAE buffer gel. NZYDNA Ladder III was used as a DNA molecular weight marker. In addition, the DNA quantity and quality were measured by reading the entire absorption spectrum (220–750 nm) with a NanoDrop ND-1000 micro-spectrophotometer and calculating DNA concentration and absorbance ratio at both 260/280 and 230/260 nm. The machine was calibrated and cleaned according to the calibration check procedure.

### PCR Amplification and Sequencing

DNA samples were diluted and equalized to 50 ng/μL. PCR reactions were performed in 50 μL reaction mixtures containing Taq 2x VWR Master Mix, 25 μL; genomic DNA, 1 μL; 10 μM primers, 2 μL (1 μL for each primer used); and ultrapure sterile water, 22 μL. The amplifications were performed using a Bio-Rad C1000 thermocycler. After a preliminary trial, primer pairs ITS1/ITS4 for the ITS region of the nuclear ribosomal RNA operon, EF1-728F/EF1-986R for partial *EF1-*α gene amplification, and Bt2a/Bt2b for partial *tub* gene amplification were selected and used in this study applying PCR conditions reported by the authors (Table 2). Amplified products were resolved by electrophoresis using the same conditions previously described. PCR products were purified using the E.Z.N.A. Cycle Pure kit (Omega) according to the manufacturer’s instructions and then sent to a commercial laboratory that performed Sanger sequencing (Eurofinsgenomics, Germany).

**TABLE 2:** Primer sets and corresponding amplification targets.

**Target region**	**Primer name**	**Primer sequence**	**Size of PCR amplicon**	**References**
β-*tub* gene	Bt2a	5′ GGT AAC CAA ATC GGT GCT TTC 3′	500 bp	Glass and Donaldson, 1995
	Bt2b	5′ ACC CTC AGT GTA GTG ACC CTT GGC 3′		
*EF 1-α* gene	EF1-728F	3′ CAT CGA GAA GTT CGA GAA GG 5′	350 bp	Carbone and Kohn, 1999
	EF1-986R	3′ TAC TTG AAG GAA CCC TTA CC 5′		
ITS	ITS1	5′ TCC GTA GGT GAA CCT GCG G 3′	600 bp	White et al., 1990
	ITS4	3′ TCC GCT TAT TGA TAT GC 5′		

### Molecular Data Analysis

Sequence files were processed using the pattern analysis software package BioEdit Sequence Alignment Editor v.7.0.5.0 (Hall, 1999). To establish the identity of the strains at the species level, combined phylogenetic analyses of the three considered loci (ITS + *tub* + *EF1-*α) were conducted against those of different species sequences selected by extensive literature review (Udayanga et al., 2011, 2014a,b, 2015; Gomes et al., 2013; Lombard et al., 2014; Huang et al., 2015; Dissanayake et al., 2017a,b; Gao et al., 2017; Santos et al., 2017a,b; Fan et al., 2018; Guarnaccia et al., 2018; Yang et al., 2018; Long et al., 2019; Manawasinghe et al., 2019; Marin-Felix et al., 2019; Ozawa et al., 2019; Zhou and Hou, 2019; Hilário et al., 2020) and retrieved from the National Center for Biotechnology Information (NCBI) database (Supplementary Table 1). Individual alignments were performed using the MUSCLE tool (Edgar, 2004) implemented in MegaX (Kumar et al., 2018). Poorly aligned positions and divergent regions were eliminated using the Gblocks v.0.91b online tool (Castresana, 2000).

In order to perform a multigene phylogeny reconstruction, datasets were concatenated using the online tool FaBox (Villesen, 2007). The most suitable substitution model was determined based on the lowest Bayesian information criterion. Maximum-likelihood trees were constructed in MegaX through 1,000 bootstrap replications (Felsenstein, 1985) based on the Tamura-Nei (Tamura and Nei, 1993) substitution model (TN93) considering non-uniformity of evolutionary rates among sites modeled using a discrete Gamma distribution (+ G) with 5 rate categories and assuming that a certain fraction of sites is evolutionarily invariable (+ I). Bayesian posterior probabilities of branches were computed in MrBayes v.3.2.7 (Ronquist et al., 2012) using settings for the best-fit model selected by the Akaike information criterion in MrModeltest v.2.4 (GTR + I + G) (Nylander, 2004). Obtained trees were edited in the iTOL v.5.6 program (Letunic and Bork, 2019).

### Data Analysis

In relation to the hazelnut growing season, different meteorological parameters were computed for four different time periods (P1: 1 January-30 April; P2: 1 May-30 June; P3: 1 July-31 July; P4: 1 August-30 August). In particular, the mean air temperature (Tm;°C) was calculated as the mean of the temperature for each period; degree day (DD;°C) was computed as the sum of the mean daily T; summation of DD (°C) was obtained by adding the DD of each period; total R (mm) was computed as the sum of daily R (mm); summation of R (mm) was obtained by adding R of each period; mean RH (RHm; RH%) was calculated as the mean of the RH for each period.

SPSS software (IBM SPSS Statistics v. 24) was used for the data analysis. Analysis of variance (ANOVA) was applied to arcsine-transformed data on fungi incidence in hazelnut kernels. Tukey’s test was used to indicate statistically significant differences between mean values. Pearson correlation analysis was run between the incidence of defective hazelnuts, assessed on 3,000 hazelnuts per orchard at full ripening, and the incidence of fungi at the same sampling time, so as between the incidence of defective nuts and meteorological parameters computed for the four time-periods.

## Results

### Sampling and Meteorological Data

Early ripening sampling occurred on July 15–20, while the full ripening sampling was performed on August 5–10.

Similar values of Tm were recorded for the same period in the different Turkish provinces, but Sakarya and Samsun commonly had Tm that was lower and Trabzon higher than the mean (Table 3). In particular, during the time period P1, a minimum DD of 691 was recorded in Sakarya, and a maximum DD of 1041 was recorded in Trabzon. For the time period P2, the minimum DD was recorded in Samsun and the maximum in Zonguldak, with values of 934 and 1077 DD, respectively, while for the time periods P3 and P4, the minimum DD was recorded in Samsun and maximum in Trabzon, with values ranging from 632 to 723 DD in the third period and from 636 to 763 DD in P4. Further, only 70 mm of rainfall were recorded during P1 in Giresun, while more than 150 mm were recorded in all the other studied locations during the same time period, with a maximum of 282 mm in Düzce. Giresun was the province with the most abundant rainfall during the entire considered period, with ΣR of 793 mm *versus* a minimum of 290 mm recorded in Zonguldak.

**TABLE 3:** Summary of meteorological data collected during 4 periods (P1-P4) from 1st January to 30th August in different Turkish provinces (Düzce, Giresun, Ordu, Sakarya, Samsun, Trabzon, and Zonguldak) in 2017.

	**Start date**	**End date**	**Mean air temperature (Tm,°C)**	**Degree Day (DD,°C)**	**Summation Degree Day (ΣDD,°C)**	**Total rainfall (R, mm)**	**Summation rainfall (ΣR, mm)**	**Mean air relative humidity (RHm,%)**
Düzce								
P1	1-Jan	30-Apr	7.1	858	858	282	282	81
P2	1-May	30-June	17.3	1068	1926	168	450	87
P3	1-July	31-July	22.2	690	2616	37	487	84
P4	1-Aug	31-Aug	22.6	700	3316	39	526	86
Giresun								
P1	1-Jan	30-Apr	7.4	888	888	70	70	70
P2	1-May	30-June	16.3	997	1885	155	225	84
P3	1-July	31-July	21.0	652	2538	239	463	84
P4	1-Aug	31-Aug	21.6	671	3209	330	793	91
Ordu								
P1	1-Jan	30-Apr	7.4	888	888	250	250	69
P2	1-May	30-June	16.4	999	1887	99	349	82
P3	1-July	31-July	21.1	655	2542	8	357	81
P4	1-Aug	31-Aug	21.9	680	3223	33	390	88
Sakarya								
P1	1-Jan	30-Apr	5.8	691	691	210	210	82
P2	1-May	30-June	16.3	994	1685	164	374	86
P3	1-July	31-July	20.6	638	2323	85	459	86
P4	1-Aug	31-Aug	20.8	645	2968	54	513	88
Samsun								
P1	1-Jan	30-Apr	6.2	747	747	206	206	70
P2	1-May	30-June	15.3	934	1682	167	373	84
P3	1-July	31-July	20.4	632	2314	16	389	82
P4	1-Aug	31-Aug	20.5	636	2949	53	443	90
Trabzon								
P1	1-Jan	30-Apr	8.7	1041	1041	273	273	72
P2	1-May	30-June	17.6	1075	2116	190	462	84
P3	1-July	31-July	23.3	723	2839	33	495	80
P4	1-Aug	31-Aug	24.6	763	3602	123	618	85
Zonguldak								
P1	1-Jan	30-Apr	7.4	883	883	155	155	75
P2	1-May	30-June	17.7	1077	1961	3	158	82
P3	1-July	31-July	22.1	686	2647	29	187	80
P4	1-Aug	31-Aug	22.0	682	3329	103	290	83

### Fungal Isolation and Identification

The results of fungal isolation are based on the analysis of 3,780 half-cut nuts and are reported here as incidence (%) of hazelnuts infected by each genus. Different fungi were isolated from plated half kernels; the prevalent fungi were *Alternaria*, *Aspergillus*, *Botryosphaeria*, *Diaporthe*, *Fusarium*, *Penicillium*, and *Pestalotiopsis.* Furthermore, *Rhizopus*, *Cladosporium*, *Trichoderma*, *Botrytis*, *Trichothecium*, and *Mucor* were also occasionally isolated, but always with incidence lower than 10%.

**FIGURE 2:**
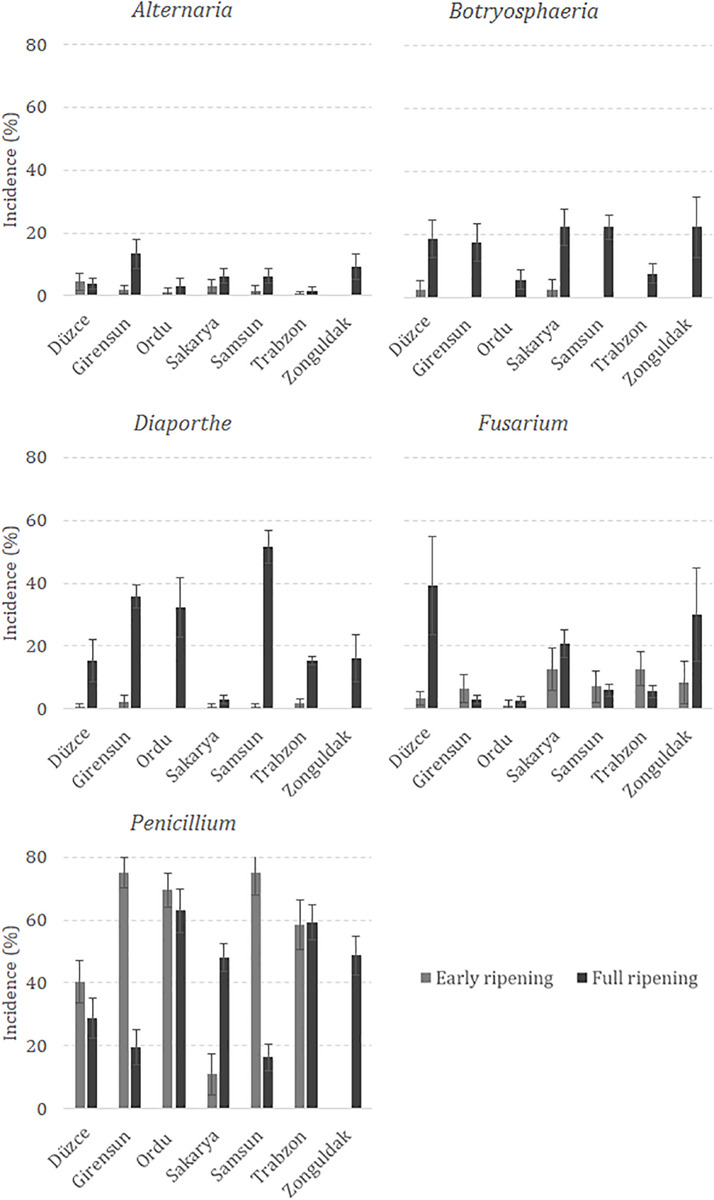
Mean incidence of *Alternaria, Botryosphaeria, Diaporthe, Fusarium* and *Penicillium* isolated from hazelnuts sampled in Düzce, Giresun, Ordu, Sakarya, Samsun, Trabzon and Zonguldak at early and full ripening.

The ANOVA was run considering fungal incidence data collected during 2 sampling dates (early and full ripening) and in 7 Turkish provinces (Düzce, Giresun, Ordu, Sakarya, Samsun, Trabzon, and Zonguldak). Hazelnut growth stage at sampling significantly affected the incidence of *Alternaria*, *Botryosphaeria*, *Diaporthe*, and *Fusarium*. In particular, the highest incidence of *Alternaria*, *Botryosphaeria*, *Diaporthe*, and *Fusarium* was observed at full ripening. *Penicillium* was the most isolated genus, followed by *Diaporthe*. Regarding the role of geographic areas, for *Alternaria* spp. significant differences were only noticed between Giresun (7.2%), Trabzon (1.1%), and Ordu (2.2%). The highest incidence of *Botryosphaeria* was observed in Düzce, Sakarya, Samsun, and Zonguldak (mean 11.2%). The hazelnut growing province with the highest incidence of *Diaporthe* spp. was Samsun (26.1%), which was not significantly different from that of Giresun (19.3%). The highest incidence of *Fusarium* was observed in Düzce (21.3%), which was significantly different from that of Giresun (4.4%), Ordu (2.0%), and Samsun (6.5%). *Penicillium* incidence ranged between 68% in Ordu and 24.4% in Zonguldak. The impact of the sampling place was not significant for *Aspergillus* and *Pestalotiopsis* spp. Furthermore, the interaction between factors significantly affected the incidence of isolated fungi except for *Aspergillus* and *Pestalotiopsis* spp. (Table 4). Minor differences were noticed compared to the impact of main factors. Regarding *Alternaria*, no increase was observed in Düzce and Trabzon between early and full ripening, while it was reported for the main factor “location”; similarly, *Fusarium* spp. decreased in Giresun, Samsun and Trabzon from early to full ripening showing an opposite behavior compared to the main factor “location”. On the contrary, *Penicillium* commonly decreased from early to full ripening, except in Sakarya, Trabzon and Zonguldak (Figure 2).

**TABLE 4:** Mean incidence of the most isolated fungal genera in hazelnuts sampled at early and full ripening in 7 Turkish provinces (Düzce, Giresun, Ordu, Sakarya, Samsun, Trabzon and Zonguldak). Growth stage and province were considered as factors in ANOVA.

	***Alternaria***		***Aspergillus***	***Botryosphaeria***		***Diaporthe***		***Fusarium***		***Penicillium***		***Pestalotiopsis***
Growth stage (A)	**		n.s.	**		**		**		n.s		n.s
Early ripening	1.8	b	1.3	0.7	b	0.9	b	7.4	b	47.1		0.3
Full ripening	6.2	a	1.2	16.5	a	24.2	a	15.3	a	40.6		0.2
Province (B)	**		n.s.	**		**		**		**		n.s.
Düzce	4.1	ab	0.9	10.4	a	8.0	cd	21.3	a	34.6	cd	0.0
Giresun	7.2	a	2.5	8.7	ab	19.3	ab	4.3	cd	46.8	bc	0.5
Ordu	2.2	b	1.0	2.8	b	15.7	bc	2.0	d	68.0	a	0.2
Sakarya	4.7	ab	0.2	12.3	a	1.9	e	16.7	ab	29.5	de	0.2
Samsun	4.0	ab	2.3	11.1	a	26.1	a	6.5	bcd	45.7	bc	0.0
Trabzon	1.1	b	1.1	3.7	bc	8.4	cd	9.2	abc	59.0	ab	0.9
Zonguldak	4.6	ab	0.6	11.1	a	8.1	cde	19.2	ab	24.4	e	0.0
Interaction												
A x B	**		n.s.	**		**		**		**		n.s.

n.s., not significant. **P ≤ 0.01; different letters define significant differences according to the Tukey test.

Regarding the incidence of defective hazelnuts, at early ripening, the total defects were ≤0.5%, while at full ripening, they ranged between 0.3 and 4.1%. At full ripening, differences were noted between the defect incidence in East (mean 3.1%) and West (mean 0.4%) provinces, with 3, 2.3, 4.1, and 3.1% incidence observed in Giresun, Ordu, Samsun, and Trabzon, respectively (East Turkey) *versus* 0.6, 0.3, and 0.2% incidence scored in Düzce, Sakarya, and Zonguldak, respectively (West Turkey). The incidence of internal defects, observed after nuts are half-cut, contributed between 15 and 45% to total defects.

Pearson correlation analysis, run between the incidence of defective hazelnuts and of fungi at full ripening, proved *Diaporthe* as the only genus positively correlated with internal (ρ = 0.86, *P* ≤ 0.01) and visible defects (ρ = 0.81, *P* ≤ 0.05, Table 5). Negative correlation was recorded for *Fusarium* (ρ = −0.81 and ρ = −0.82, *P* ≤ 0.05 for internal and visible defects, respectively). Although not significant (*P* ≥ 0.05), positive related trend with both internal (ρ = 0.32) and visible (ρ = 0.46) defects was found for *Aspergillus* (Table 5). Regarding meteorological parameters, no significant correlation was found, neither for defects nor for fungi incidence.

**TABLE 5:** Pearson correlation analysis, run between incidence of hazelnuts defects (internal and total) and incidence of fungi at full ripening.

**Province**	**Location**	**Internal defects^+^ (%)**	**Total defects (%)**	***Alternaria***	***Aspergillus***	***Botryosphaeria***	***Diaporthe***	***Fusarium***	***Penicillium***	***Pestalotiopsis***
Düzce	West	0.2	0.6	3.7	0.0	18.5	15.2	39.3	28.9	0.0
Giresun	East	0.9	3.0	13.3	3.3	17.4	35.9	3.0	19.6	0.0
Ordu	East	1.0	2.3	3.0	0.7	5.6	32.2	2.6	63.0	0.4
Sakarya	West	0.1	0.3	6.3	0.4	22.2	3.0	20.7	48.1	0.4
Samsun	East	1.5	4.1	6.3	0.7	22.2	51.5	5.9	16.3	0.0
Trabzon	East	0.8	3.1	1.5	1.9	7.4	15.2	5.6	59.3	0.7
Zonguldak	West	0.0	0.3	9.3	1.1	22.2	16.1	30.0	48.7	0.0
		Correlation internal defects		–0.07	0.32	–0.35	0.86**	−0.81*	–0.30	0.05
		Correlation total defects		–0.03	0.46	–0.33	0.81*	−0.82*	–0.33	0.13

^+^Internal defects are intended as those visible after kernels half-cut; **P ≤ 0.01; *P ≤ 0.05.

### *Diaporthe* Phylogenetic Analysis

In order to reach sub-genus identification of the *Diaporthe* strains isolated from Turkish, Caucasian, and Italian hazelnuts, phylogenetic analysis was first performed against a set of 280 *Diaporthe* spp. strains selected from the literature (Supplementary Table 1) and representing a comprehensive overview of this genus. As shown in Supplementary Figure 1, the phylogeny is highly complex, with genetic distances (branch lengths, not shown) and bootstrap support values being generally low, which is the reason why it was difficult to establish clades or species complexes in this dataset.

**FIGURE 3:**
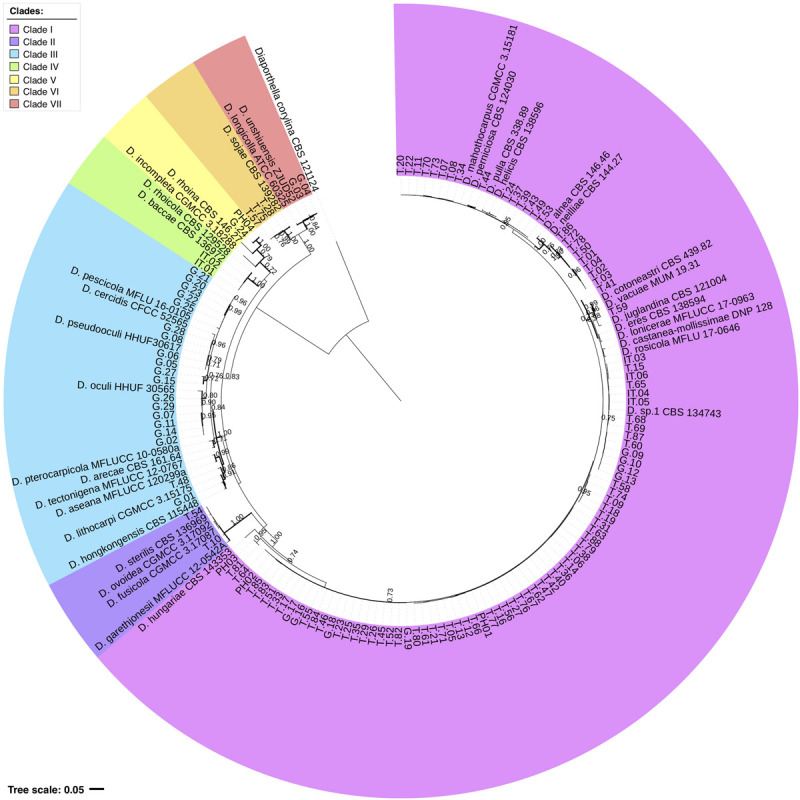
Combined phylogeny for the ITS, *EF1-*α, and *tub* sequence data of the 125 strains isolated from hazelnuts with other *Diaporthe* strains detailed in Supplementary Table 1. Diaporthella *corylina* CBS 121124^T^ was used as the outgroup. Selected model: TN93 + G + I. The percentage of trees in which the associated taxa cluster together in the bootstrap test (1,000 replicates) is shown above the branches. Bold branches are supported with ≥0.95 Bayesian posterior probability. The tree is drawn to scale with branch lengths measured in the number of substitutions per site. All positions with less than 95% site coverage were eliminated. The final dataset included 162 nucleotide sequences and a total of 981 positions. The tree branch topology is shown in more detail in Supplementary Figure 2.

In order to reduce the dataset complexity and improve grouping support, a second phylogenetic tree was constructed using a set of closely related reference strains that were selected based on the grouping patterns observed in the broader phylogenetic analysis. In this case (Figure 3), it was possible to define 7 well-supported clades (bootstrap values ≥70%; Bayesian posterior probability ≥95%). Two of them, clade I and III, included 91.2% of the total number of strains, with geographic origin being a factor supporting the diversity observed. Clade I contained 76% of the total number of analyzed strains and was mainly composed of Turkish strains (82 out of 95 in the clade), with 9 Caucasian and 4 Italian strains. However, clade III included 15.2% of the analyzed samples and was mainly composed of Caucasian strains (18 out of 19) and one from Turkey. The remaining clades are most likely to represent occasional and opportunistic species.

## Discussion

Because of kernel defects, hazelnut yield loss and market value decrease are observed worldwide, and studies with the aim of understanding the origin of such defects are crucial (Garrone and Vacchetti, 1994). Several *Diaporthe* species have been reported as causing diseases in different types of nuts (Osmonalieva et al., 2000; Teviotdale et al., 2002; Rhouma et al., 2008; Diogo et al., 2010; Gramaje et al., 2012; Chen and Morgan, 2014; Fan et al., 2015, 2018; Annesi et al., 2016; Gao et al., 2017; Pscheidt and Ocamb, 2017; Yang et al., 2018; Eichmeier et al., 2020), including hazelnuts (Guerrero and Pérez, 2013; Guerrero et al., 2014, 2019), but only very recently, a comprehensive four-year study performed in the Caucasus region confirmed the role of this genus in causing external brown spots on nuts and also those observed after kernel half-cut (Battilani et al., 2018). In the present study, fungi associated with defective hazelnuts from Turkey, the leading hazelnut producer worldwide, were investigated to eventually confirm these results.

Several fungal genera were isolated in this survey; they may have been influenced by the hazelnut varieties, as previously stated for *D. rudis* (Pscheidt et al., 2019), but this factor cannot be discussed in this study because a mix of different hazelnut varieties were grown in the sampled orchards. Among the isolated fungi, the key role of *Diaporthe* spp. in hazelnut defects was confirmed by the observed significant differences of its incidence in kernels produced in the considered Turkish provinces. Furthermore, *Diaporthe* spp. is the only fungal genus positively correlated with hazelnut defects, both those on the surface and those visible after kernel half-cut. The latter are of particular interest from a commercial point of view, as internal defects cannot be found using optical sorters. Therefore, it is not easy to discharge kernels with internal defects from the commercial product. Moreover, *Aspergillus* spp. was positively related to hazelnut defects, even if not significantly. This should be studied deeper in order to find out eventual interaction between *Diaporthe* and related defects with *Aspergillus;* this genus includes well-known fungi that can lead to severe economical and health impacts due to mycotoxin production (Ozay et al., 2008; Kabak, 2016; Houshyarfard and Javadi, 2018). The most isolated genus was *Penicillium*, in agreement with Pscheidt et al. (2019), but relations between this fungus and defects were not found. Nevertheless, *Penicillium* also includes mycotoxin producing fungi and merit to be studied deeper for possible health issue implications.

The highest incidence of *Diaporthe* spp. was recorded in the provinces located in Eastern Turkey, where the highest incidence of defects was also registered. In particular, the incidence at full ripening was the highest in Samsun (51.5%), the province where the highest incidence of total defects in nuts (4.1%) was observed. The lowest incidence of *Diaporthe* was recorded in Western Turkey provinces (mean 11.4%), with the lowest total defect incidence (mean 0.4%). *Diaporthe* spp. incidence increased from early to full ripening, in agreement with a previous study (Battilani et al., 2018), and that closely corresponds to the increase in observed defects that occurs during the hazelnut ripening stage. However, defect incidence was comparable with the data from years of low defect incidence reported for Caucasian hazelnuts (incidence of 1.1–3.3%), while up to 14.3% incidence was reported in 2016 (Battilani et al., 2018). Further, the correlation with rainfall was not confirmed. The lower incidence of defective nuts compared to the Caucasian hazelnuts and the limited variation between orchards (0.25–4.13%) was insufficient to highlight the role of this or other meteorological parameters.

Although this study aimed to realize identification at the species level for the in-depth characterization of *Diaporthe*, this was not possible for a majority of species. As discussed by Fan et al. (2018), increased numbers of species in the alignment led to reduced accuracy in species separation, an effect that is partially dependent on the number of analyzed loci. Here, only 4 strains could be confidently grouped with two known species (*D. unshiuensis* and *D. pseudooculi*). The remaining strains were either grouped with more than one reference strain or formed clusters (with different degrees of branching support) with no known species included. Based on the phylogenetic analysis results (Figure 3), the 125 hazelnut strains were grouped into 7 clades, and some included members of known species complexes. Clade I included type strains of the *D. eres* species complex (*D. alnea*, *D. eres*, *D. helicis*, *D neilliae*, and *D. pulla*), and clades VI and VII included species from the *D. sojae* species complex (*D. longicolla* and *D. sojae*).

It is interesting to note that several Caucasian strains in clade III were related to *D. oculi* and *D. pseudooculi*, which are species that were initially described as human pathogens and are phylogenetically related to the *D. arecae* species complex (Ozawa et al., 2019). Clade III includes *D. arecae* and *D. hongkongensis*, which are also members of the *D. arecae* species complex (Long et al., 2019). Despite the limited power of our dataset, it is interesting to see that the sample grouping and relation to known species is mostly maintained in both the extended (Supplementary Figure 1) and reduced (Figure 3) analysis. This is of particular importance in clades I and III, as they include a large number of strains that cannot be classified at the species level, and some of them are possible taxonomic novelty candidates.

When comparing the hazelnut taxonomic composition with that of other nuts (mainly walnuts and almonds), it is interesting to see that each study reports slightly different *Diaporthe* species present in the considered matrix, with *D. rostrata*, *D. amygdali*, and *D. eres* being more recurrent. *D. foeniculina* has been reported in sweet chestnuts from Italy and hazelnut kernels from Chile (Annesi et al., 2016; Guerrero et al., 2019), *D. rudis* in hazelnut kernels from Oregon, United States (Pscheidt et al., 2019), and *D. australafricana* from Chilean hazelnuts (Guerrero and Pérez, 2013); among these species, only *D. eres* was found to be related to the hazelnut strains studied here.

The observation of poorly supported or non-monophyletic clades is a common situation within *Diaporthe*, probably due to ITS heterogeneity within species complexes (Udayanga et al., 2014b; Huang et al., 2015; Guarnaccia et al., 2018). This situation can be improved if a multi-locus sequences analysis approach is considered, particularly with the inclusion of *EF1-*α or *tub* gene regions (Udayanga et al., 2012a), as applied in this study. Therefore, species and species complex limits need to be carefully defined, and for that reason, no species-level classifications were performed at this point. Recent studies have successfully identified new species based on the same three loci used here (ITS, *EF1-*α, and *tub*) (Lesuthu et al., 2019; Zapata et al., 2020). Nevertheless, some studies used four or more loci (Udayanga et al., 2014a, b, 2015; Zhou and Hou, 2019), and a study from Santos et al. (2017a) showed that the most optimal *Diaporthe* species separation occurred when five loci were simultaneously used.

In conclusion, the present study shows that despite the heterogeneous nature of the hazelnut cultivable mycobiota, *Diaporthe* spp. are the only fungal species strongly associated with both internal and external defects in hazelnut kernels. A comparison of strains from different geographic origins showed that different species were responsible for similar symptoms (clade I *versus* clade III). Finally, a majority of Turkish *Diaporthe* strains are related to *D. eres*, a well-known plant pathogen that has been previously reported in *C. avellana*. Future studies focusing on improved molecular-based species classification, particularly of those strains belonging to clades I and III, will prove to be valuable to clarify their role as causative agents of hazelnut defects, assist with developing control strategies, and increase the quality and quantity of available product that meets market requirements.

## Data Availability Statement

The datasets presented in this study can be found in online repositories. The names of the repository/repositories and accession number(s) can be found below: https://www.ncbi.nlm.nih.gov/search/all/?term=, MT613733–MT613857; https://www.ncbi.nlm.nih.gov/search/all/?term=, MT849830–MT849954; https://www.ncbi.nlm.nih.gov/search/all/?term=, MT840703–MT840825.

## Author Contributions

PB: conceptualization and supervision. PB, RA, GCh, NL, and CSa: experiment design. RA, CSa, and CSo: experiment management. PB, RA, NL, and CSa: data analysis. CSa and RA: phylogenetic analysis. RA and CSa: original manuscript preparation. PB, GCh, NL, GCa, NS, and CSo: review and editing. PB, GCh, NL, GCa, and NS: funding acquisition. All authors contributed to the article and approved the submitted version.

## Conflict of Interest

GCa and NS were employed by the company Soremartec Italia S.r.l. The remaining authors declare that the research was conducted in the absence of any commercial or financial relationships that could be construed as a potential conflict of interest.

## Footnotes

1 http://www.statista.com

2 http://www.fao.org/3/x4484e/x4484e03.htm

3 https://www.unece.org

4 www.fao.org

## Supplementary Material

Supplementary Figure 1 Tree branch topology representation of the combined phylogeny for ITS, *EF1-*α, and *tub* sequence data of the 125 strains isolated from hazelnuts with other *Diaporthe* strains selected from the literature and detailed in Supplementary Table 1. *Diaporthella corylina* CBS 121124^T^ was used as the outgroup. Selected model: TN93 + G + I. The percentage of trees in which the associated taxa cluster together in the bootstrap test (1,000 replicates) is shown above the branches. All positions with less than 90% site coverage were eliminated. The final dataset included 406 nucleotide sequences and a total of 684 positions.

Supplementary Figure 2 Tree branch topology representation of the tree presented in Figure 3. Combined phylogeny for ITS, *EF1-*α, and *tub* sequence data of the 125 strains isolated from hazelnuts with other *Diaporthe* strains selected from the results presented in Supplementary Figure 1 and detailed in Supplementary Table 1. *Diaporthella corylina* CBS 121124^T^ was used as the outgroup. Selected model: TN93 + G + I. The percentage of trees in which the associated taxa cluster together in the bootstrap test (1,000 replicates) is shown above the branches. Bold branches are supported with ≥0.95 Bayesian posterior probability. All positions with less than 95% site coverage were eliminated. The final dataset included 162 nucleotide sequences and a total of 981 positions.

Supplementary Table 1 List of strains used for the phylogenetic analyses with the corresponding ITS, *EF1-*α, and *tub* GenBank sequence accession numbers. The list includes a set of *Diaporthe* species selected from the literature and the 125 hazelnut strains amplified in this study.
