# Supplementary material for: Molecular Characterization of Diaporthe Species Associated With Hazelnut Defects
Source: Front Plant Sci. 2020 Dec 11;11:611655. doi: 10.3389/fpls.2020.611655 (PMC7759530; doi:10.3389/fpls.2020.611655)
Supplement: Supplementary Figure 1 — Tree branch topology representation of the combined phylogeny for ITS, EF1-α, and tub sequence data of the 125 strains isolated from hazelnuts with other Diaporthe strains selected from the literature and detailed in Supplementary Table 1. Diaporthella corylina CBS 121124T was used as the outgroup. Selected model: TN93 + G + I. The percentage of trees in which the associated taxa cluster together in the bootstrap test (1,000 replicates) is shown above the branches. All positions with less than 90% site coverage were eliminated. The final dataset included 406 nucleotide sequences and a total of 684 positions. [file Data_Sheet_1.PDF]

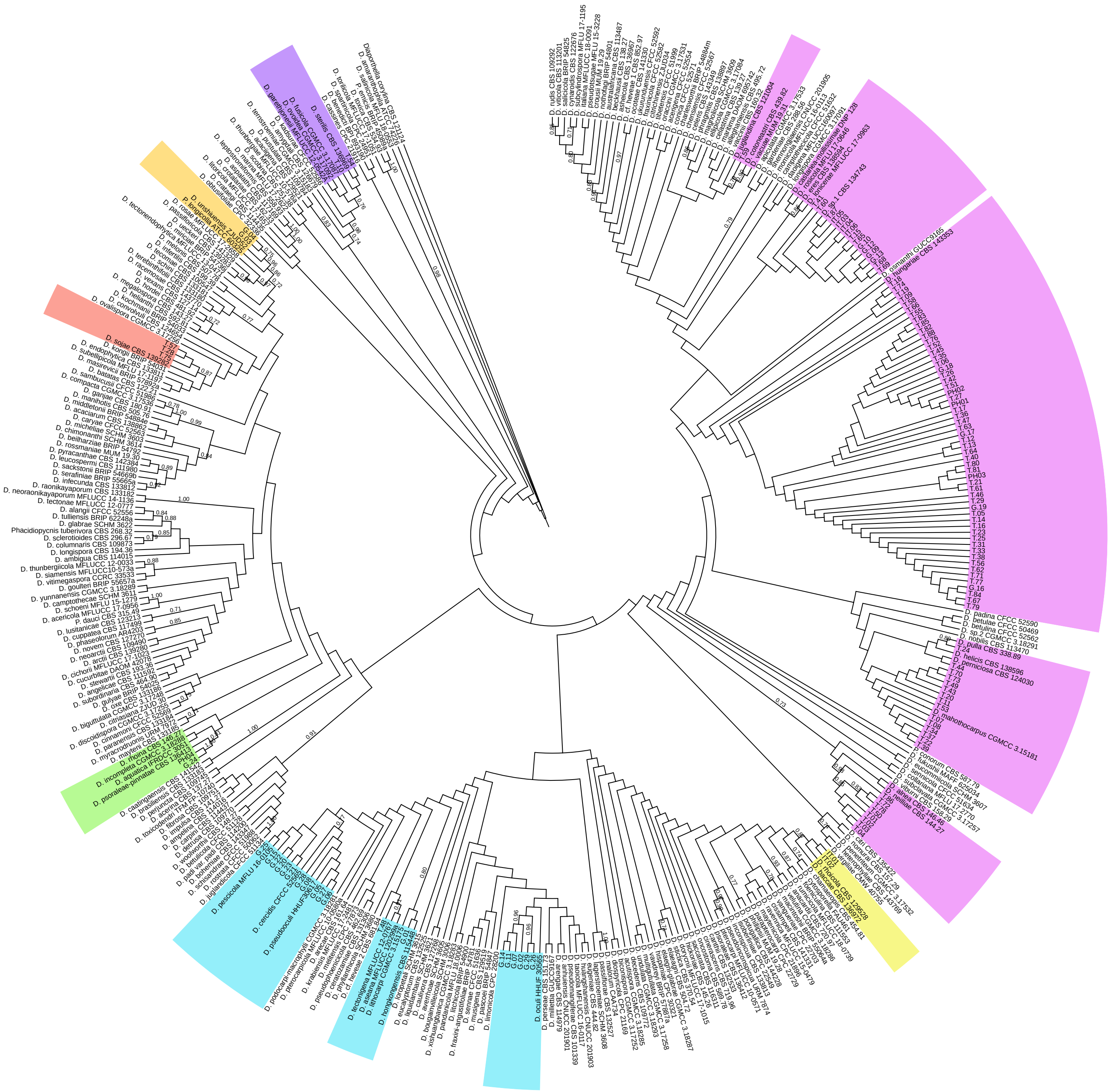

Supplementary Figure 1. Tree branch topology representation of the combined phylogeny for ITS, *EF1-α*, and *tub* sequence data of the 125 strains isolated from hazelnuts with other *Diaporthe* strains selected from the literature and detailed in Supplementary Table 1. *Diaporthella corylina* CBS 121124^{T} was used as the outgroup. Selected model: TN93+G+I. The percentage of trees in which the associated taxa cluster together in the bootstrap test (1,000 replicates) is shown above the branches. All positions with less than 90% site coverage were eliminated. The final dataset included 406 nucleotide sequences and a total of 684 positions.

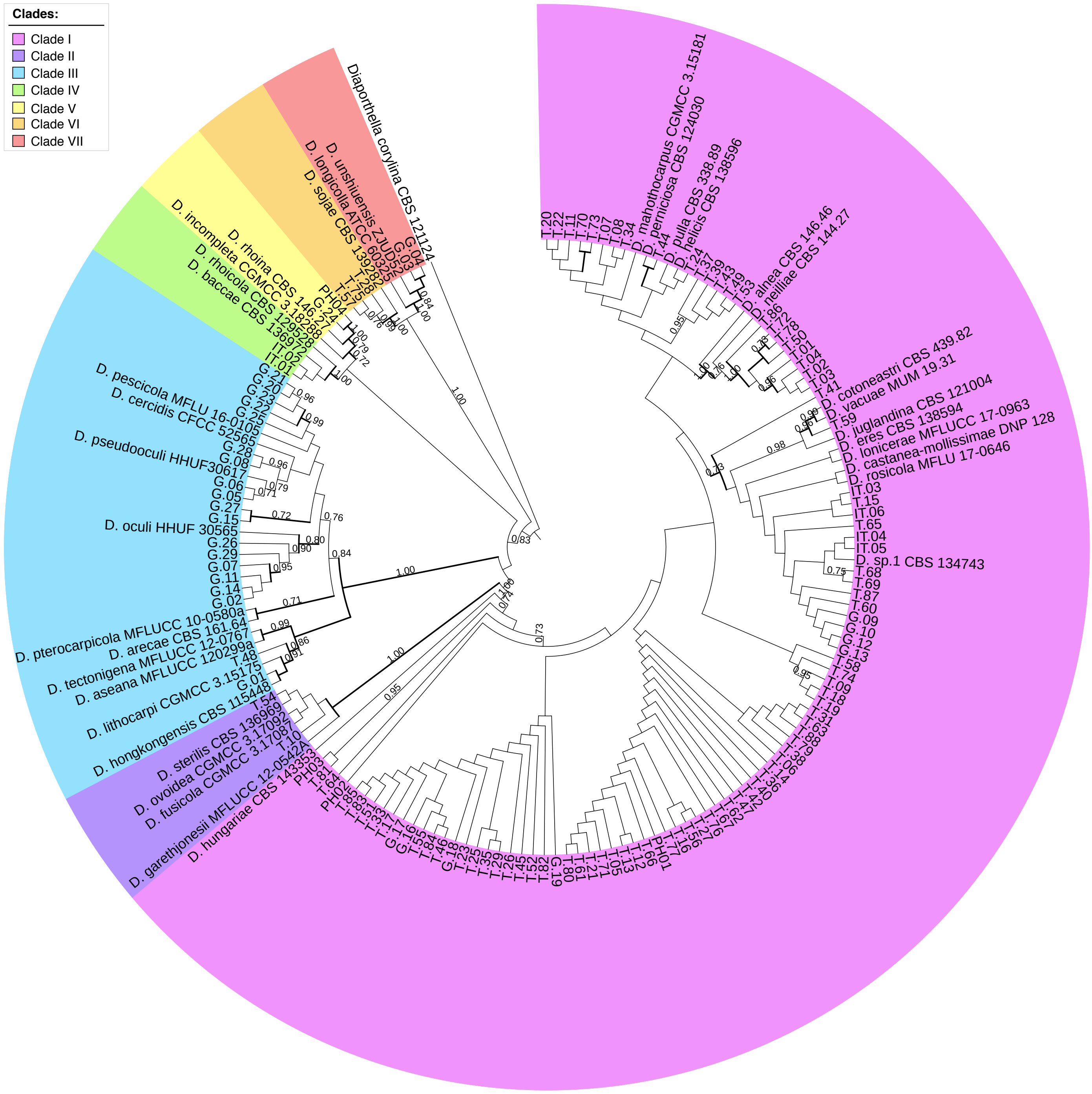

Supplementary Figure 2. Tree branch topology representation of the tree presented in Figure 3. Combined phylogeny for ITS, *EF1-α*, and *tub* sequence data of the 125 strains isolated from hazelnuts with other *Diaporthe* strains selected from the results presented in Supplementary Figure 1 and detailed in Supplementary Table 1. *Diaporthella corylina* CBS 121124^{T} was used as the outgroup. Selected model: TN93+G+I. The percentage of trees in which the associated taxa cluster together in the bootstrap test (1,000 replicates) is shown above the branches. Bold branches are supported with ≥ 0.95 Bayesian posterior probability. All positions with less than 95% site coverage were eliminated. The final dataset included 162 nucleotide sequences and a total of 981 positions.
